# Two billion registered students affected by stereotyped educational environments: an analysis of gender-based color bias

**DOI:** 10.1057/s41599-022-01220-6

**Published:** 2022-07-30

**Authors:** Jário Santos, Ig Bittencourt, Marcelo Reis, Geiser Chalco, Seiji Isotani

**Affiliations:** 1 Institute of Mathematics and Computer Science, University of São Paulo (ICMC-USP), CEP: 13566-590 São Carlos, SP Brazil; 2 Institute of Computer Science, University of Alagoas (UFAL), Maceió, Brazil, CEP: 57072-970 Maceió, AL Brazil; 3 Computer Science Center (C3), Federal University of Rio Grande (FURG), CEP: 96203-900 Rio Grande, RS Brazil

**Keywords:** Cultural and media studies, Environmental studies, Education, Science, technology and society, Psychology

## Abstract

According to the literature, educational technologies present several learning benefits to promote online education. However, there are several associated challenges, and some studies illustrate the limitations in elaborating educational technologies, called Design limitations. This aspect is responsible for unleashing various issues in the learning process, such as gender inequality, creating adverse effects on cognitive, motivational, and behavioral mediators, which opposes the fifth UN’s Sustainable Development Goal. Therefore, many studies notice the harmful effects of stereotypes in educational technologies. These effects can be included in the design, like colors or other stereotyped elements, or how the activity is conducted. Based on this, the present study aimed to verify the predominance of color bias in educational technologies available on the WEB. This study developed a computational solution to calculate male and female color bias in the available educational technology web pages. The results suggest the prevalence of the development of educational technologies with a male color bias, with an imbalance among genders, without adequate customization for age groups. Furthermore, some environments, such as Computer Science, present a higher color bias for men when compared to women. Despite both scales being independent, results indicated interesting evidence of a substantial prevalence of colors associated with the male scale. According to the literature, this may be associated with dropout and lack of interest in female students, especially in sciences, technology, engineering, and mathematics domains.

## Introduction

Studies debated various features contained in educational technologies, including benefits, challenges, and strategies of online education (Bailey and Lee, 2020). The current scenario caused by the Covid-19 pandemic has expanded the niche, and the need for educational technologies in order to improve teaching-learning processes and styles (Dhawan, 2020). Moreover, according to Dhawan (2020), this promotes the growth of educational technologies, providing suggestions to academic institutions as to how to deal with the challenges associated with online learning, training, and further education to develop students’ independence by improving digital skills to an academic level (Jackman et al., 2021). This also provides support for teachers in the students’ inquiries (Goudeau et al., 2021), prevent cheating (Li et al., 2021), and increase engagement. Especially when it is not possible to have direct contact with students in the classroom due to the current pandemic scenario and when procedures need to be adjusted to manage academic subjects and teaching resources (Gillett-Swan, 2017; Hafeez et al., 2021). Alongside the issues mentioned above, opportunities also arise, such as possibilities to develop new teaching methods (Almossa and Alzahrani, 2022), learning support from artificial intelligence interactions (Pataranutaporn et al., 2021), improving access to education in rural zones, and study hours flexibility (Adedoyin and Soykan, 2020; Vlachopoulos, 2020).

Nevertheless, some studies illustrate the limitations in elaborating educational technologies (Schöbel et al., 2020), frequently called Design limitations. That is, attributes that may be used for an adequate elaboration of educational technologies (Klock et al., 2015), in a manner in which technologies become more customized, in aspects such as (i) age; (ii) gender; (iii) motivations; and, lastly, (iv) student profile. The latter is necessary because students of different profiles may interact differently with the teaching platforms (Espinoza et al., 2020). Students can be encouraged by different attributes such as videos, quizzes, experience points (Geving, 2007; Hill, 2006). This use of attributes may help prevent inequalities, such as (i) some students learn more than others; (ii) lower engagement in certain student groups (Forman et al., 2020); (iii) students of opposite genders not being able to understand the exact issue (Pedro et al., 2015); and (iv) high evasion rates per student group.

This stereotype limitation in educational technologies is responsible for unleashing many problems (Darling-Hammond et al., 2020). Learning inequalities lead to various adverse effects (Pennington et al., 2016) such as cognitive mechanisms mediated by cognitive load (Croizet et al., 2004; Kith et al., 2022) leading to a decrease in cognitive performance due to the effects of stereotype threat. The decrease in work memory due to stereotype-related distractions (Doncel-García et al., 2022; Johns et al., 2008; Schmader and Johns, 2003; Turner and Engle, 1989). These may also lead to mind-wandering, with studies reporting an increase in stereotype-related thoughts and concerns when those were triggered in priming tasks (Brown Morris, 2022; Rydell et al., 2014; VanLandingham et al., 2021). Additionally, motivational mechanisms mediated by achievement goals showed that high difficulty activities induced apprehension (Chalabaev et al., 2008; Elliot and Church, 1997; Seo and Lee, 2021). Moreover, dejection in groups in uneven scenarios was related to lower performance (Hoeve, 2022; Keller and Dauenheimer, 2003). Lastly, behavioral mechanisms mediated by anxiety may affect the use of gamified technologies with gender discrepancies (Albuquerque et al., 2017; Grier et al., 2022). Also, self-efficacy was reported to have a significant impact on performance and motivation when participants are presented with stereotyped cues (Maddux, 1993; Navarro et al., 2022; Schunk, 1989). Such issues are objects to studies in a strand of the literature called Stereotype Threat, which consists of an individual’s exacerbated concern of being evaluated based on a negative stereotype (Myers et al., 2014). This stereotype is characterized by the incidence of patterns prone to please a certain group (Lippmann, 1946). This preference may lead to better results among individuals of target groups when compared to those of impaired groups, as is evidenced by learning performance indicators (Hsu et al., 2022), which was reported to be due to effects brought by cognitive (Kith et al., 2022; Schmader et al., 2008), and behavioral mechanisms (Gerstenberg et al., 2012). Anxiety is a potential mediator in this process, promoting a significant impact on learning performance, and is frequently related to stereotype threat.

Several studies noticed adverse effects of stereotypes in educational technologies, whether these are included in the design through stereotyped colors, elements, and texts or during the execution of an activity. By using elements of stereotyped design, Chang et al. (2019) presented evidence that interactions in educational platforms with stereotyped Avatars cause a decrease in women’s learning performances when interacting with these Avatars with male-dominated design. Albuquerque et al. (2017) proposed an experiment to analyze colors in gender-stereotyped gamified environments in order to assess if gender-related colors influenced students’ anxiety levels. The study used blue for male-stereotyped environments, lilac for female-stereotyped, and gray for the control setting. Results concluded that changes in women’s anxiety levels were more significant than those of men while using male-stereotyped technology. Nonetheless, stereotype threat may be centered not only around attributes such as colors composing educational technologies but also the interactions with the elements themselves. Christy and Fox (2014) discussed the configuration of ranking tables and texts in scoreboards regarding stereotype threat. According to the authors, there was evidence that women, when in a setup with a female-dominant ranking table, presented lower performances in the mathematics test when compared to women in a setup with a male-dominant ranking table.

Three aspects of stereotype threat (text, interactions, and colors) are considered in educational technologies. The textual analysis depends on specific language nuances (AlBadani et al., 2022). This type of analysis would require universal linguistic models able to handle at least most world languages. Therefore, besides a large amount of data, it would also require high computational power for training and realignment for each language (Taghizadeh and Faili, 2022) including regional variations. On the other hand, to observe stereotype effects on users’ interactions would require data user logs in every single system, as well as users to follow a standardized data collection (Nguyen et al., 2022), which would exponentially increase the task’s complexity. However, using colors and their biases, we can focus on just a few aspects of the design of educational technologies (Albuquerque et al., 2017; Kuo et al., 2022). Therefore, the data collection and analysis complexity can be reduced by applying the tools to collect color data.

Motivated by the adverse effects of stereotype threat in educational technologies, this study aimed to verify the existence of prevalence in the level of color preferences (a.k.a. color bias) in educational technologies. Additionally, this study aimed to present how color design is used, considering specific aspects such as the type of technology, context, and target audience, regarding gender and age. Given the availability of information on the web, we chose to focus on four types of educational technologies: (i) CMS—content management systems; (ii) RLE—remote learning environments (AVA—Virtual Learning Environments); (iii) Gamified Environments; and lastly (iv) MOOCs—Massive open online courses, used as teaching technologies of seven teaching subjects: (1) Business, (2) Computer Science, (3) Languages, (4) Math, (5) Multidisciplinary, (6) Programming and (7) Sciences. In order to evaluate the color bias in educational technologies and the prevalence of color preferences, the following research questions were formulated: The gender category was divided into male and female only.

1. What is the color preference (color-bias) in educational technology design?
2. What is the color preference (color-bias) present in educational technologies design according to the teaching subjects (context)?
3. What is the color preference (color-bias) concerning the colors present in the design according to the types of educational technologies?
4. What is the color preference (color-bias) present in educational technologies design according to the age range of the target group?

This article is organized in the following manner: section two describes the theoretical framework and the related studies, presenting stereotype threats, the metrics used, and the gamified educational settings of this study. Section three presents the proposal and describes the tools used in this study. Section four presents and discusses the results. Lastly, in section five, the study conclusions are addressed.

## Theoretical framework

The following section presents a brief literature review with the main concepts and theories adopted as a basis for the present study.

### Stereotype threat

Stereotype, in its conceptualization, has a Greek origin which means (“stereo” —rigid; “typos”—impression). The concept was used to represent a form of impression manufactured in metallic parts for the production of books during the 18th century (Del Boca and Ashmore, 1980). In 1981, Walter Lippman aggregated a new conceptualization of the word, defining it as previously constituted mental representations, which somehow influenced the ability to conduct activities.

Stereotypes became known as beliefs, resulting in a prejudiced judgment regarding a specific target, and became an object of study for social psychology. Such studies observed the intellectual complexity linked to the development of activities when comparing performance (Yzerbyt et al., 1997). It was noted that when the stereotype unleashes a negative sense, the individual may suffer a series of issues, which may affect psychological mediators-namely, cognitive, behavioral, and motivational mechanisms. When the effect is perceived, the individual who is affected enters a state of threat (Pennington et al., 2016).

Stereotype threat consists of negative effects on an individual’s performance in a certain task (Shapiro and Neuberg, 2007). Several studies in the literature observe the effects of stereotype threats in social groups (identity groups and non-identity groups, Gonzales et al., 2002; Martiny et al., 2012). These studies often identified decreased performance when participants of minority groups faced stereotyped environments. Some studies investigated and discussed the stereotype threat effect (Flore and Wicherts, 2015; Lamont et al., 2015; Nguyen and Ryan, 2008; Shewach et al., 2019) and its correlation with performance (Lewis Jr and Michalak, 2019). Through the development of activities to evaluate the performance of minority groups while performing a task and developing stereotyped scenarios to simulate and verify stereotype threat effects. The attributes related to stereotype threat comprise elements such as colors in the design of educational technologies.

Effects caused by stereotypes and performance decreases are present in much of educational technology’s attributes. Based on that, studies discussed educational technologies that may favor a group. Nonetheless, when the technology presents gender stereotypes, this may greatly disfavor the learning process of the other group. Gender stereotyped educational technologies are currently an essential subject of study associating possible causes and effects. For instance, Albuquerque et al. (2017) presented a study on the impact of stereotype threat and anxiety on the performance of a logic test. Nonetheless, the subject still raises many questions due to curious results. Christy and Fox (2014) presented evidence that women when in a setup with a female-dominated ranking table presented lower performances in the mathematics test when compared to women in a setup with a male-dominated ranking table.

### Educational Technologies and color-bias

Colors are understood as objects that have three components (Ibraheem et al., 2012): (i) hue—the combination that can be made by using shades of red, green, and blue (RGB); (ii) saturation—the attenuation degree of a specific color, i.e., its intensity; (iii) brightness - an attribute that defines the characteristic of light emission, that is, the state of giving out or reflecting light.

However, a color can be much more than an element of design: it may be related to different feelings, emotions, and desires (Rider, 2010) and related to how human brains can capture it. Understanding the whole process of assimilation, from its activation to how the perception can influence human behavior through colors, is the object of the study of color psychology (Singh and Srivastava, 2011; Whitfield and Whiltshire, 1990).

Based upon this relation, understanding the primary feelings and emotions aroused through the perception (Webster, 1996) of a specific color, it is possible to elaborate a correlation with the meaning of the information which a particular color may convey. The literature presents various studies relating feelings originating from colors, ranging from tranquility to the impression of something hazardous (Simmons, 2011).

In order to understand further the color relations with emotions and behavior, studies considered individual color preferences as a way to relate one’s emotion to his/hers current mental state. Through that, it was possible to observe the changes in color emotional response throughout the years, as well correlate color preferences with gender (Cunningham and Macrae, 2011) or according to age group (Pope et al., 2012). Some studies (Best et al., 1975; Clark and Clark, 1940; Duckitt et al., 1999) observed that colors are also associated with trend biases: positive-white and negative-black, which can be strongly linked to documents that represent cultural and racial groups.

Studies also reported that the relationships between colors and human beings could be further extended into characteristics that involve the perception of color based on gender. Hill (2002) analyzed the relationship between skin colors and the meaning attached to it. In this study, results suggested that men related the skin with female characteristics based on the color tone associated with the skin. Furthermore, Jakobsdóttir et al. (1994) presented significant differences between color preferences between men and women and discussed the guidelines for developing graphics (images) that should be used. This was also pointed out by Volman and van Eck (2001), who considered color as a possible leveling attribute for gender equity in educational technologies.

Although the literature does not directly address existing color bias in educational technologies, it has no shortage of studies that show that color bias can directly influence some elements or mediators, whether in the design (Albuquerque et al., 2017; Richard, 2017) or in the educational scope itself (Brandon et al., 2021). The literature presents evidence that although colors are strongly related to children’s future choices, stereotyped elements belonging to the same gender can influence them even more (Karniol, 2011). Furthermore, studies also reported that graphic elements are generally perceived differently by men and women, which allows questions about differences in learning in educational technologies to arise (Chanlin, 2001).

### The issue of gender in educational technologies

Currently, many studies present factors that should be better explored in educational technologies. Although several factors such as age group, ethnic group, and culture influence inclusion parameters, gender remain one of the easiest to control and study due to its number of classes. Studies reported distinct styles in the learning process between men and women, as well as choices by disciplines more suited based on these profiles (Steffens and Jelenec, 2011). Comparisons can identify trends, such as mentioned by Steffens et al. (2010), Vuletich et al. (2020): women prefer disciplines directed towards the elaboration of content for personal growth, while men tend to logic and reasoning.

Among other gender-related aspects, the subject of stereotypes and educational technologies and how it has the potential to favor a group while disfavoring or hindering the learning of other has been approached by scholars in recent years. Albuquerque et al. (2017) presented a study on the negative impact of stereotype threat and increased anxiety in the performance in logic tests. Moreover, Lee and Nass (2012) showed that, in educational technologies, the females tend to be fewer concerns associated with stereotypes and presented overall better performances in math tests while cooperating instead of when competing.

Components included in the design exert influence over results as well as Chang et al. (2019) presented evidence that women who had their learning performance impaired while interacting with male instructors used non-verbal sexist behavior. Furthermore, Christy and Fox (2014) reported that women, when in a setup with a ranking table that is female-dominant, showed lower performance in the math tests when compared to women in a setup with a male-dominant ranking table. However, the moderator’s avatar did not significantly impact women’s performance in the same conditions.

### Related works

Subjects like safety and moral standards have been associated with many arguments considering the World Wide Web since its early days. Using the large amount of data that has been produced on the internet in recent years Sagiroglu and Sinanc (2013), researches highlighted ethical aspects (Ogbuke et al., 2022), privacy (Saura et al., 2021) and security (Díaz et al., 2022) of the immense amount of data. Other studies pointed to data bias in applications with artificial intelligence and natural language processing (Caliskan et al., 2017; Hellman, 2020; Kleinberg et al., 2018; Mitchell et al., 2021; Pessach and Shmueli, 2020). Moreover, some authors observed flaws in algorithmic fairness in education (Kizilcec and Lee, 2020) and further discussed challenges to accessing this data for research, considering ethics and justice.

**Fig. 1:**
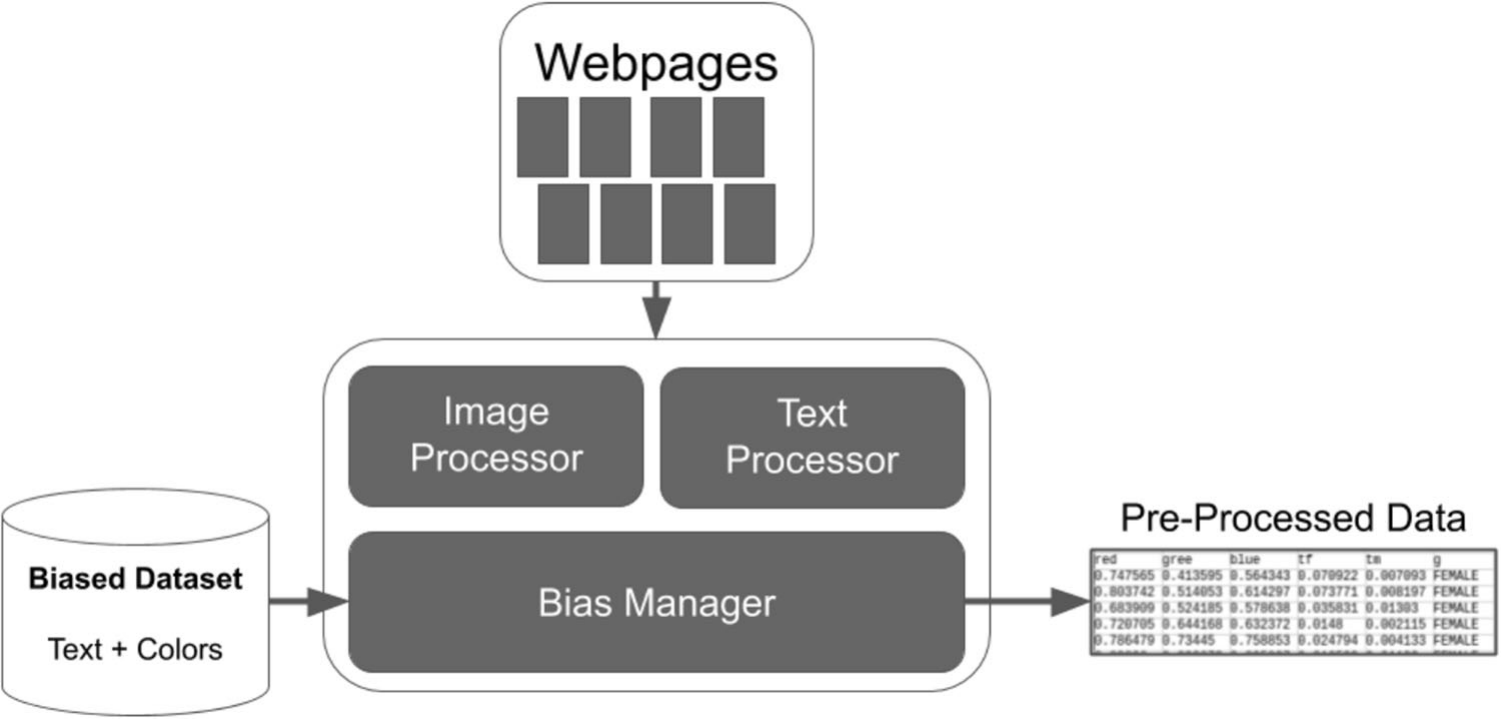
Silva et al. (2019) approach. The figure represents the data collection process carried out by Silva et al. (2019) in images and texts on websites.

Silva et al. (2019) suggested a possible solution with a supervised learning approach to detect gender stereotypes in online educational technologies. Similarly, Silva et al. (2019) proposed the implementation of a data collection technology on websites available on the WEB to extract gender bias from the contents present on its pages. In order to construct these datasets, the authors proposed a search that included website contexts not restricted to educational, although in this study, they only analyzed educational sites. Furthermore, the authors proposed a computational solution based on image and text processors and a bias management system (Fig. 1).

The limitations found in Silva et al. (2019) proposal can be seen in two dimensions: Technical and Ethical, the Ethical dimension being the most critical. In the Technical dimension, it is observed that the collected data are only from the main page of the corresponding educational technology. Therefore, explicit stereotypes might be present on other pages of the same technology, generating inaccuracies in calculating color bias in the sample. Additionally, no smoothing in the pixel calculation was observed in this study. The principle behind this smoothing process is to allow calculations of page similarity based on RGB standards, considering averages only. In turn, the ethical dimension is of utmost importance due to morality issues and from a legal point of view: the authors referred to the data collection process without considering permission criteria and which pages are accessible for collection or not. Thus, works that discuss, for example, areas correlated with ethical principles also need to conduct studies that follow these same ethical standards, and studies should present summarized comparisons with the main research topics of each proposal (Table 1).

**Table 1:** Related work comparison.

Study	Technical dimension				Ethical dimension	
	Color	Text	Static files	Pages	Data protection	Regarding ethics
Silva et al. (2019)	X	X	–	One	X	–
Present study	X	–	X	All*	X	Robots.txt and Meta-tags

X = It satisfies the requirement; – = It does not satisfy the requirement; * = in accordance with access permissions.

## Methodology

The current study investigates the presence of color bias existing in educational technologies. Furthermore, based on the assumption of its existence, observe the impact of this bias on diverse target audiences and their respective age groups. Thus, observe if there are color differences in technologies by respective types and context.

**Fig. 2:**
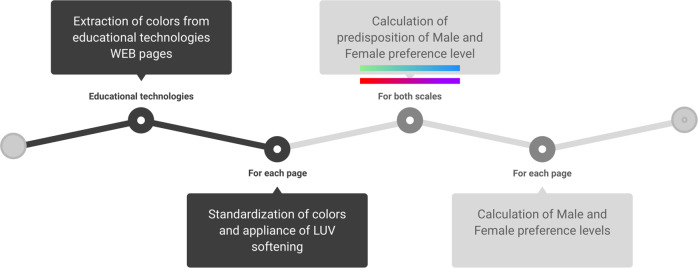
Execution flow of the bias calculation solution. The figure presents the execution flow of the computational application to calculate the final color bias level of the educational technologies considered in this work.

The character of the present study is observational and intends to *detect and measure the color preference level in educational technologies, considering male and female gender*. In order to answer our research questions, a computer solution was created to estimate the color preference level among genders through a process developed to identify colors in educational technologies (Fig. 2). The developed algorithm receives the Uniform Resource Locator (URL) of a given educational technology and identifies the colors contained on the main page. This tool also access colors on secondary pages of the respective educational technology. The representation of similarities between colors is not adequate colors are composed of three shades. Therefore, to calculate similarities between them, it was necessary to perform two treatments: (i) standardization—which consists of applying a standard between collected colors, varying tonality between 0 and 255—this assures the averages of the RGB components of the processed colors. Moreover, the standardization allows calculating a resulting color, simplifying interpretation. Furthermore, this process was necessary to normalize all the pixels on a page, highlight the most present colors for analysis, and discard rare colors that could have affected the results. In other words, only the most frequent colors were considered for the analysis, thus resulting in color equalization (Yongan et al., 2012; Zhong et al., 2008); (ii) LUV softening—consists of applying a vector decomposition, consisting of the more accurate vector representation (Kakooei and Baleghi, 2022) between two colors. In other words, with LUV softening, it is possible to calculate how close two colors are in terms of similarity, creating a more semantic representation of the colors in a vector space. The LUV softening effect produces more pragmatic colors, which place them closer to human visual perception and facilitate identification (Zhang et al., 2020). Furthermore, LUV softening was the base calculation of smoothing for constructing the male and female scales in each educational technologies page and classifying them according to how they are perceived by the human eye, considering color segmentation in its hue, saturation, and brightness. After these two processes (standardization and softening), the predisposition of existing colors was calculated in male and female scales based on gender-related color perception. The construction of these gender-based scales considered the color range that best fits current preference profiles. According to Fulcher and Hayes (2018), Yeung and Wong (2018), the color range of pink and purple was a preference for females, while blue and green for males. Kodžoman et al. (2022), Kuo et al. (2022) also presented color ranges of pink and blue as colors with preference highest levels among women and men, respectively. Based on these classic scales and color ranges, the male scale taken as a basis was proposed by Silver and Ferrante (1995), presenting color preferences for masculine colors in shades of green and blue. For the female scale, it was taken as a basis, the scale proposed by Hallock (2003), where women’s color preference for shades of red, pink, and purple is displayed. Lastly, with the colors and scales arranged, the calculation of the male and female preference levels for each page composing the educational technology is carried out through the cosine.

### Materials

The computational solution in this study is composed of processing modules described in further detail below. Moreover, the source code collected data and statistical analysis are available in an available online repository^1^ for access and evaluation. Overall, a total of six processing modules were used, as follows:

- *Encase and anonymity of technology links*: The algorithm receives as input a file called ’urls.txt’ containing links to educational technologies. Afterward, it applies a hashing function to encrypt the access link. Given this, the algorithm creates a new spreadsheet (dictionary.csv) with a list of URLs with encrypted data to organize the samples that will be collected in the next step;
- *Collection of pages links*: The algorithm accesses the spreadsheet file, accessing links of educational technologies homepages, retrieving all the pages contained in that technology and that have access permission (more details in section “Ethics on data collection procedure”), then, a new spreadsheet file (pages.csv) is created containing the pages associated with the educational technology being processed;
- *Pages screenshot*: the algorithm access the “pages.csv” spreadsheet file scanning page by page, taking a screenshot, and saving it;
- *Pixels collection and normalization*: The algorithm randomly scans each of the screenshot images, collecting a total of 3000 colored pixels above the white color tone. White-colored pages were discarded by the tool for further analysis. Nonetheless, these pages were recorded in a file (’whitepageslist.txt’). In order to guarantee the average of the colors in the Red-Green-Blue (RGB) pattern, the algorithm applied pixel normalization to colored/non-white pages. The RGB model was chosen as a standard broadly used, and due to its compatibility with all color systems adopted for educational technologies’ development (Olsson, 2014);
- *LUV smoothing*: This step transformed the RGB pattern into a LUV decomposition to assure the representation of colors with greater accuracy, especially considering the variety of color shades to serve as input to the next step;
- *Similarity calculation*: The distance between the colors of the scales was calculated with the colors extracted from sampled pages to calculate the degree of similarity between the male and female scales. The cosine was the metric chosen for representing more accurately, following the metrics established by Tao et al. (2017) and Techapanurak et al. (2019, 2020). Cosine calculation further allows the measurement of the distance between two values and considers directionality, as blue and red would present opposite directions in the scale. It is worth remembering that both color scales are standardized with LUV smoothing leveling similarities calculation. As the final step, similarity values were aggregated by page and, thus, the values of respective levels of female and male preference.

#### Ethics on data collection procedure

The present study used data mining concepts on the Web, taking into account authorization of which files can be accessed and collected through permissions files (like robots.txt and meta-tags, Van Wel and Royakkers, 2004), such as Robots Exclusion Protocol (REP). These establish standards for whether to access data and which part of this data is permitted by query robots available on the Web, comprising ethical norms and principles and the use of information that does not require approved access.

Therefore, the robots.txt file was checked to verify access permissions for each site’s web page (i.e., educational technologies). The file follows a structure of which agents and which pages can get accessed. Generally, an asterisk indicates that any computer agent (robot) will not be able to consult or access the respective page, which was listed in the body of the file. Some specifications allow robots to access certain content, such as Facebook or Twitter agents that can have access to profile content.

**Fig. 3:**
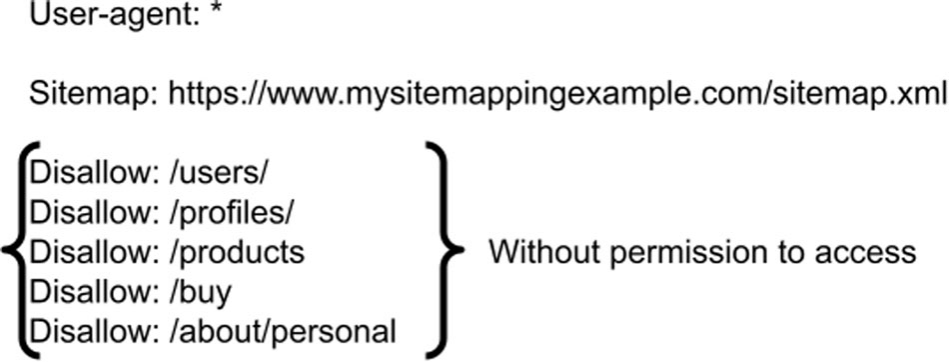
Robots.txt example. The figure presents a mapping structure with access permissions and its pages. The mapping is responsible for locating all technology pages, following its permission or restriction of access with the pages.

Pages like users, profiles, products, buy, and about/personal have access restrictions for any agent. However, pages such as “index” or “about” may have granted access to robots.txt example files. Figure 3 shows a file example with the specific pages without permissions to access.

The literature concerned with such ethical concepts follows this convention (robots.txt or meta-tags) from web data mining for open linked data (Oren et al., 2008), web content mining (Költringer and Dickinger, 2015), mining learners participating data in learning environments (Kop et al., 2011). All of these ethical concepts were taken into account for the construction of the data of this study.

**Fig. 4:**
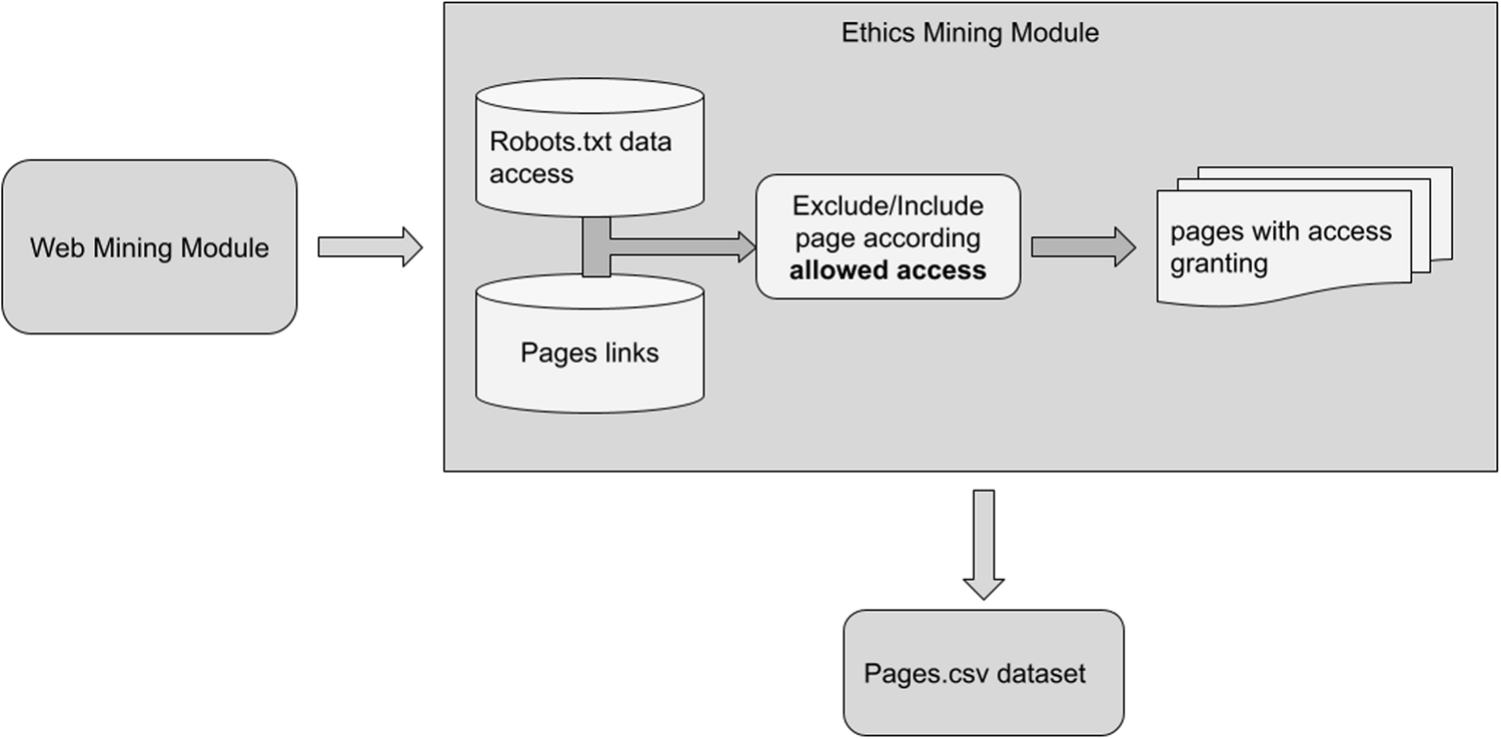
Responsible web mining data collection procedure. The figure presents the data extraction process, following ethical concepts for access and availability. All the dataset construction and access to the pages of educational technologies were analyzed with access release.

The process of link extraction and sampling for building the dataset (“pages.csv”) used in this study was developed in three stages: (i) web mining; (ii) ethical mining; and (iii) data collection (Fig. 4)

- Web mining module: The first stage consisted in accessing main sites, also called Indexes or Homepages. This step checked the presence and access granted by Robots.txt files. All links referenced on this page were verified according to such restrictions and access permissions in the second stage;
- Ethical mining module: The second step applied access filters to what may or may not be consulted on pages that could be accessed later. All inclusions and deletions were performed by consulting the Robots.txt file, following the standards of each site. Links with access restrictions were deleted, and links with access permission passed to the next step to build the dataset;
- Data collection: Links with access permissions were stored in a file called pages.csv, with privacy and anonymity of information. Once stored, the links were encapsulated and encoded in string hash, which hid any category of the relation of the data collected with the respective site.

### Data and descriptive analysis

This study conducted a manual search for educational technologies between August and September 2021. A total of 88 technologies were considered, indexed each by its respective access link (Tables 2 and 3). However, as 15 of these presented access restrictions and specific permissions, thus, 73 educational technologies were considered, and data from 3136 pages were collected.

**Table 2:** List of educational technologies (part 1).

List	
https://web.digitalinnovation.one/home	https://studio.code.org/courses
https://pt.khanacademy.org/	https://thehuxley.com/
https://www.codecademy.com	https://www.respondeai.com.br/
https://www.coursera.org/	https://www.udemy.com/
https://www.arcademics.com/	https://www.brainpop.com/
http://www.cookie.com/	https://www.dimensionu.com/dimu/home/home.aspx
https://www.ipracticemath.com/	https://www.thinkport.org/index.html
https://www.mangahigh.com/en-us/	https://education.minecraft.net/
https://skoolbo.com/	https://www.spellingcity.com/
https://www.sumdog.com/us/	http://www.thetimetribe.com/
http://www.zoowhiz.com/	https://www.codegoat.org/
https://wonderville.org/	https://www.socrative.com/plans/
https://kahoot.com/	http://playbrighter.com/
https://www.classcraft.com/	https://home.breakoutedu.com/
https://quizizz.com/	https://www.gimkit.com/
https://www.classdojo.com/	https://www.knowre.com/
https://virtonomics.com/	https://www.duolingo.com/
http://classrealm.com/	https://ed.ted.com/
https://www.blinkist.com/	https://www.memrise.com/
https://business.busuu.com/education	https://www.sololearn.com/
https://kultivi.com/	https://www.alura.com.br/
https://avance.eyeduc.com/	https://rocketseat.com.br/
https://www.ev.org.br/#	https://www.stoodi.com.br/
http://programae.org.br/	https://gamearkos.com.br/
https://www.educacross.com.br/	http://educaland.com.br/
https://enemgame.com.br/	https://goeduca.com/
https://www.mangahigh.com/	https://play.schoolking.com.br/
https://inspark.education/	https://www.best.edu.au/
https://adaptivemechanics.edu.au/	https://www.dreambox.com/
https://www.wileyplus.com/	https://scootpad.com/
https://www.knewton.com/	https://geekiegames.geekie.com.br/
https://ingreso.ceibal.edu.uy/login	www.conecturma.com.br
www.arvoredelivros.com.br	www.dreamshaper.com/pt

**Table 3:** List of educational technologies (part 2).

List	
https://www.doodlemaths.com.br/index.php	www.escolaemrede.com.br
www.geekie.com.br	www.imaginakids.com.br
www.mlearn.com.br	www.p2s.me
https://air.MatematicandoEducation	www.qranio.com
www.kiduca.com.br	www.domlexia.com.br
https://www.beetools.com.br/	https://com.manabuacademy.manabuacademy
https://www.voceaprendeagora.com/	https://www.codebuddy.com.br/
http://www.educar30.com.br/	https://www.aulapp.com.br/
http://www.clickideia.com.br/	https://tutormundi.com/
https://estudologia.com.br/	https://focanavaga.com.br/
https://jovensgenios.com/	http://www.mapra.com.br/

Besides the access links for these educational technologies, other information was also extracted manually, such as type of technology, teaching subject, users’ numbers, and age. This data was available either on “about us” links or in available reports by the educational technology itself. Therefore, it was possible to map four types of technologies manually: (i) CMS—content management systems; (ii) RLE—remote learning environments (AVA—Virtual Learning Environments); (iii) Gamified Environments; and lastly, (iv) Massive open online courses (MOOCs), divided in seven themes (Business, Computer Science, Languages, Math, Multidisciplinary, Programming, and Sciences). Moreover, the ages according to the target audience that was informed by the technologies. This primary data analysis revealed a total “impact” of 2,494,082,054 users (registered students) in these educational technologies.

In order to understand the data in general terms and describe general statistical analysis, the data was divided into two strands (Table 4). The first strand is related to understanding the data and organizing it for further analyses (Table 5). It was observed from this data analysis a high outlier interference, mainly for Skewness and Kurtosis values. The second strand presented data considering measures of trend and locality with Winsorized variants. In this manner, the values would be less impacted by the presence of outliers. The means provided evidence of high values belonging to the male scale, indicating a mild male preference. Furthermore, the standard error and M-estimator presented values that indicate the ability to generalize the data to reality and its surroundings, respectively.

**Table 4:** Data description of the extracted main pages.

	L	U	V	Female L.	Male L.
*n* = 73, pages = 3136			impact = 2.494.082.054		
Min.	0.102	0.105	0.064	−0.234	0.371
Max.	1.000	1.000	1.000	0.431	0.923
Mean	0.581	0.5388	0.488	0.148	0.809
Skewness	0.042	0.344	0.551	−0.288	−1.961
Kurtosis	2.300	2.353	2.504	3.147	8.164
*Winsorized description*					
Winsorized mean	0.581	0.539	0.486	0.147	0.819
Winsorized mean SE	0.028	0.028	0.032	0.015	0.009
Median	0.563	0.510	0.468	0.140	0.837
Winsorized variance	0.023	0.024	0.025	0.005	0.002
M-Estimator	0.581	0.529	0.464	0.153	0.822
M-Estimator SE	0.027	0.030	0.031	0.015	0.010

**Table 5:** Data description for technologies by type.

	L	U	V	Female L.	Male L.
*ava*					
*n* = 7			impact = 2.52%		
Winsorized mean	0.525	0.517	0.467	0.223	0.811
Winsorized mean SE	0.096	0.092	0.101	0.048	0.032
Median	0.573	0.567	0.467	0.224	0.839
Winsorized variance	0.036	0.022	0.012	0.004	0.002
M-Estimator	0.536	0.533	0.440	0.222	0.828
M-Estimator SE	0.110	0.087	0.088	0.047	0.032
*gamified environment*					
*n* = 49			impact = 86.77%		
Winsorized mean	0.598	0.559	0.511	0.132	0.813
Winsorized mean SE	0.036	0.036	0.041	0.018	0.013
Median	0.563	0.514	0.478	0.124	0.845
Winsorized variance	0.029	0.032	0.037	0.004	0.004
M-Estimator	0.596	0.549	0.486	0.140	0.817
M-Estimator SE	0.037	0.039	0.045	0.016	0.015
*cms*					
*n* = 16			impact = 7.64%		
Winsorized mean	0.552	0.469	0.421	0.162	0.832
Winsorized mean SE	0.048	0.050	0.055	0.038	0.015
Median	0.526	0.460	0.383	0.161	0.821
Winsorized variance	0.022	0.015	0.022	0.011	0.001
M-Estimator	0.550	0.469	0.418	0.162	0.830
M-Estimator SE	0.047	0.054	0.070	0.035	0.017
*mooc*					
*n* = 1			impact = 3.04%		
Winsorized mean	0.639	0.602	0.564	0.155	0.829
Median	0.639	0.602	0.564	0.155	0.829

*ava* virtual learning environments, *cms* content management systems, *mooc* massive open online courses.

Therefore, by observing data description and characteristics, this study opted for robust statistical methods to analyze the results. This is due to the large number of issues reported by the literature (Mair and Wilcox, 2020), especially when there are violations of data normality. Evaluating the color preference level, or rather, bias, was used in the one-way comparison of multiple trimmed groups means statistic test as an alternative to the simple Analysis of Variance (ANOVA). Regarding male and female color preference scales belonging to the same subject evaluation, these two scales were estimated in each technology and considered related groups. Therefore, we used Yuen’s trimmed mean t-test in this analysis due to its robustness for two dependent groups. The Winsorized Correlation test calculated current correlation levels between the male and female scales. Since its use is familiar to the Person correlation, it adds robust effects to the tests (Mair and Wilcox, 2020).

In the present study, the *impact* is the number of users who, in some form, are impacted by using the educational technologies considered in this evaluation (Table 4). The information related to such metrics was extracted from the educational technologies pages or was contained in documents and records available on the WEB. It is relevant to highlight that some researchers considered at least one year of data showing the amount of educational technologies users. However, values remained extremely high despite this outdated information. Nonetheless, the total amount of users under impact is more than *2 Billion* people. In some manner, individuals made use of these platforms for acquiring knowledge, whether for training or learning new content.

The context class was elaborated, considering the activities and courses the technology in question offers. It is relevant to point out that a technology belonging to *multidisciplinary* contexts must contain more than one specific teaching subject. However, it is noted that technology of the *multidisciplinary* context could contain the minority contexts classified with a single sampling only (*n* = 1). It is also vital to note that in this analysis, the *computer science* and *business* contexts had only one technology integrating the group. In contrast, most technologies tend to diverse contexts, mainly towards independent learning of a discipline or course. Regarding the *languages* context, technologies that focused on teaching languages speech or writing as mechanisms for literacy were considered. When referring to STEM^2^ fields, there was a total of 19 technologies. Despite comprising only one technology of the sample, *computer science* showed a high male bias level. Moreover, this differed from the *programming* context because the specialty of the technology is turned towards disciplines composing computer science, whereas *programming* is only centered around the art of programming.

While observing the *impact*, as expected, technologies of multiple subjects technologies presented the highest number of users. Nevertheless, an intriguing fact is that even when adding educational technologies of STEM focus, despite constituting a representative majority when compared to *languages*, the impact provided by STEM was inferior, summing 6.372%, with a difference of almost 20% between these contexts. Such an effect can suggest a considerably low demand for courses in this category.

The technologies belonging to the *gamified environment* type possessed the highest representativeness, with a total of *49* (63%) out of the *73* educational technologies. Furthermore, it was the group of technologies that presented the higher impact. One possible explanation may be that gamified technologies have become more prominent in recent years due to game elements and characteristics, which aggregate engagement and playfulness in the learning process.

**Fig. 5:**
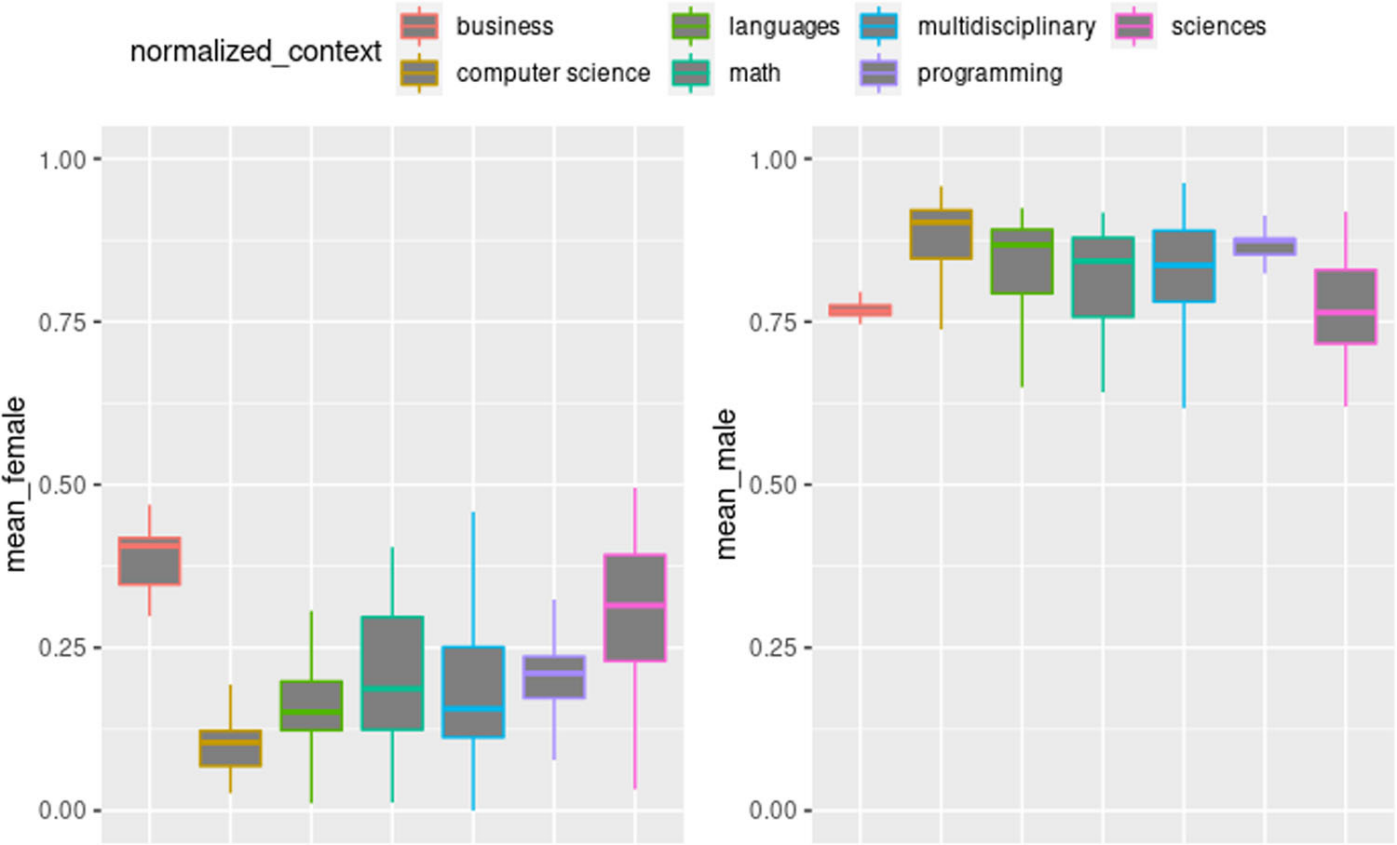
Variation of the preference levels by context. The figure presents the technologies with their respective contexts. The figure on the left side presents the layout of colors belonging to the feminine scale, while the figure on the right side presents the disposition of colors for the masculine scale. It is possible to observe that they all have a high male bias regardless of the context.

The descriptive data helps to understand the gender-based differences related to preference level by context and reveals differences and variations among male and female color scales (Fig. 5). It is important to emphasize the expected low variations due to single sampling in *computer science* and *business* contexts. However, an opposite correlation is noted in behavior between female and male preference scales. In most cases, the mean values of the female and male scales tend to be presented in the opposite direction. In the *sciences* context, it is observed a mean of higher values for the female scale, whereas, for the male scale, there is mild evidence that it is the contextual modality with the lowest mean.

**Fig. 6:**
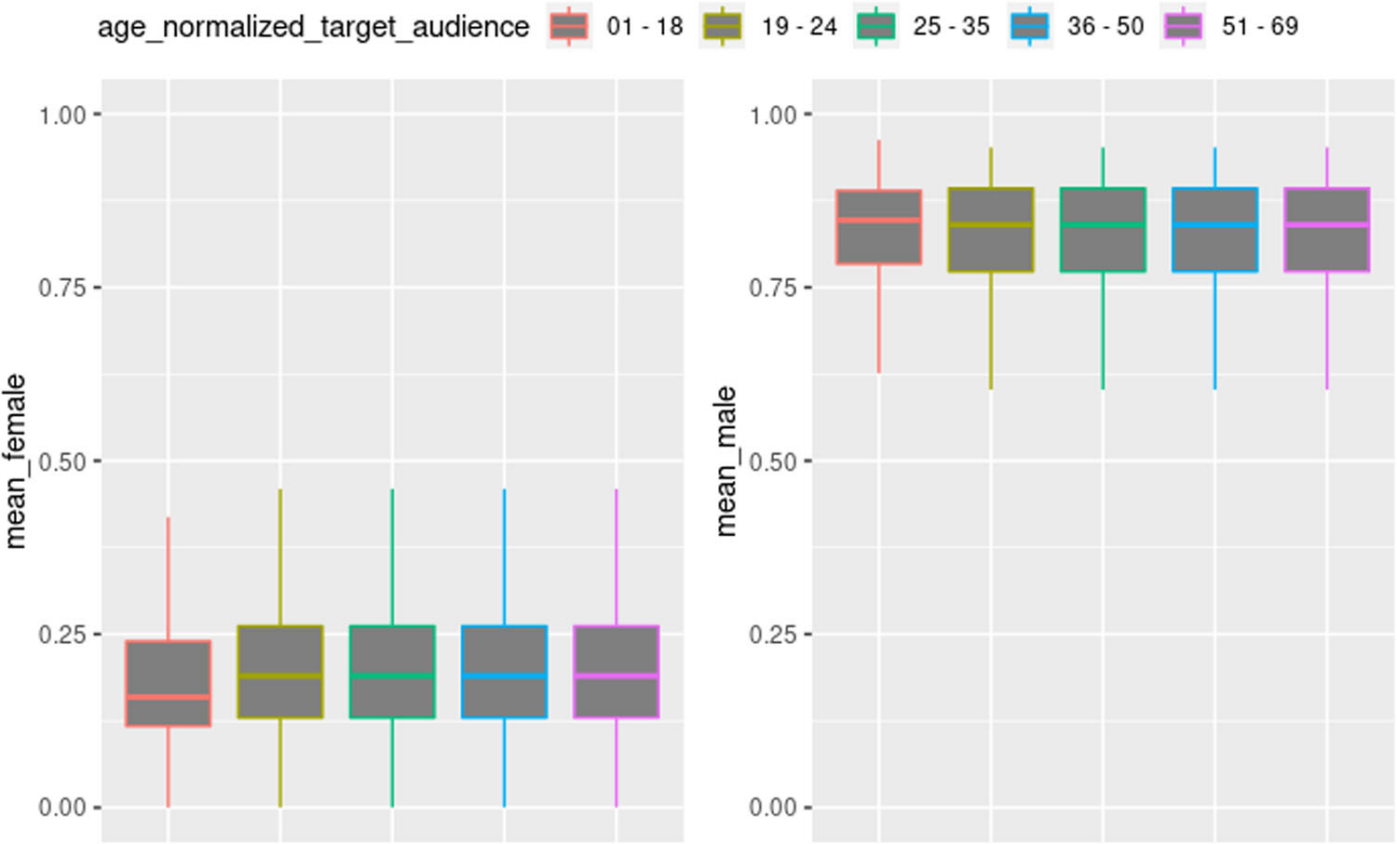
Variation of preference levels by age. The figure presents the technologies with their respective age groups. The figure on the left side presents the layout of colors belonging to the feminine scale, while the figure on the right side presents the disposition of colors for the masculine scale. It is possible to observe that they all have a high male bias regardless of the age groups.

Figure 6 presents the variation between the preference levels with target group variation. For all age groups, the male scale level is observed as higher. However, in the female scale boxplots, the medians evidence differences between them, while the male scales pattern is practically unchanged, with little variability in the median. An intriguing fact is the *6–17* boxplot, which despite having a minimum value and first quartile lower than the remaining values, the correlated boxplot in the female scale does not present an opposite effect, differing from the behavior observed in the variation of scale levels by context.

**Fig. 7:**
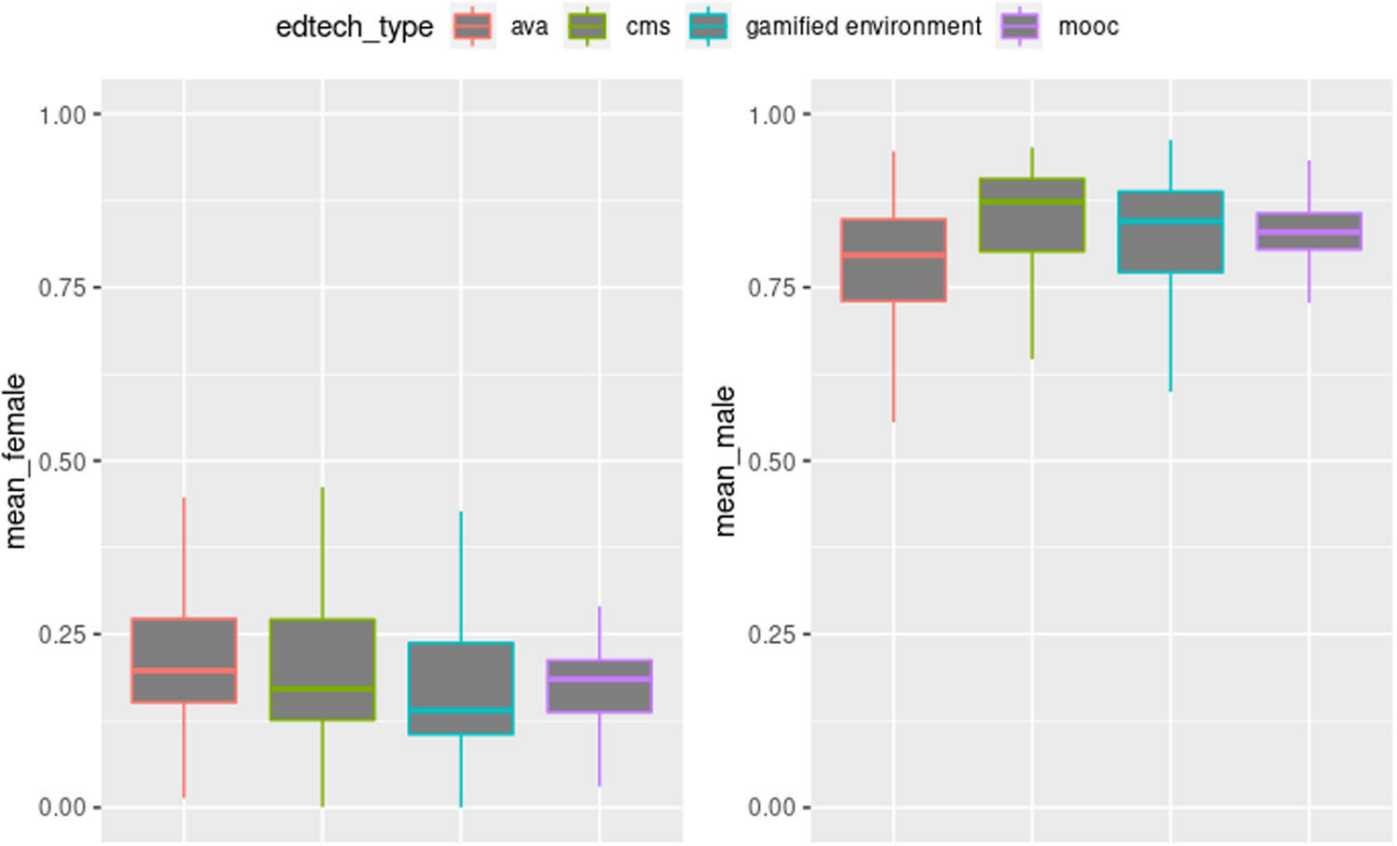
Variation of preference levels by technology type. The figure presents the technologies with their respective technology types. The figure on the left side presents the layout of colors belonging to the feminine scale, while the figure on the right side presents the disposition of colors for the masculine scale. It is possible to observe that they all have a high male bias regardless of the technology types.

The preference levels of female and male scales under the technology type show that male scales presented a low variation between medians (Fig. 7). In contrast, the *cms* type possesses a higher variability for the levels in the female scale. However, boxplots’ behavior still presents a total predominance for the male gender in these technologies, as aforementioned.

## Results

The analysis was segmented into two parts to facilitate results interpretation. The first part is related to evaluating the impact of color bias data only through the main pages belonging to educational technologies. The second part evaluated the combination of pages of each technology to understand the relationship between bias levels and their respective pages, adjusted to context, target audience, and age group, providing a deeper analysis.

### Research Question 1 (Color-bias)

Concerning the color bias in a descriptive analysis, the collected data presented different standards. Significant *p*-value for data belonging to a non-standard distribution confirm this (Table 6). The *p*-values are significant for the *B* measures, even with *W* close to 1, and *Male L*., with *W* a little further from 1. Therefore, for a more compressed analysis, tests adopted were used for the robust analysis, and transformations in the final scales could be applied for softening and standard testing. However, the development of machine learning models was used to avoid losing power and size of the effect and ensure a reliable scale for future analyses.

**Table 6:** Standard checking for the main pages’ data.

	L	U	V	Female L.	Male L.
Shapiro–Wilk (*p*-value)	0.279	0.093	0.004	0.539	<0.01
Shapiro–Wilk (W)	0.979	0.971	0.947	0.985	0.825

Results of the comparison between the calculated male and female preference levels in each technology were organized with trimming levels and reliability levels, considering preference bias and effect size (Table 7). The comparison was made through three adjustments of *trimming level* of adjusted mean values: (i) 10%; (ii) 20% and, lastly, (iii) 30%. The results showed that the male bias level is always higher than the female in the technologies evaluated in this experiment. Beyond a high effect size, degrees of freedom (df) indicate the number of ways or dimensions in which the preference levels can move without violating the restrictions, therefore, continuing to have a significant result.

**Table 7:** Preference bias of main pages with different trimming levels.

Male vs. female	Trimming level (%)	Reliability level (%)	*p*-value	df	Effect size (%)
Male greater	10	95	<0.01	58	96
Male greater	20	95	<0.01	44	96
Male greater	30	95	<0.01	30	74

In order to understand comparisons between the quantiles 3, observe the reliability interval, and the behavior of the relationship between the two preference levels (male and female), confidence intervals were listed (Table 8). Each interval was organized for each quantile, with their respective significance values. Based on the obtained values, it was possible to observe a reduction of a significant effect between quantiles.

**Table 8:** Effects of reduction by quantiles and reliability intervals in color preferences of educational technologies pages.

q	CI Low	CI Up	p-crit	p-valor
0.25	0.639	0.748	0.050	<0.01
0.50	0.636	0.726	0.025	<0.01
0.75	0.589	0.688	0.016	<0.01

**Fig. 8:**
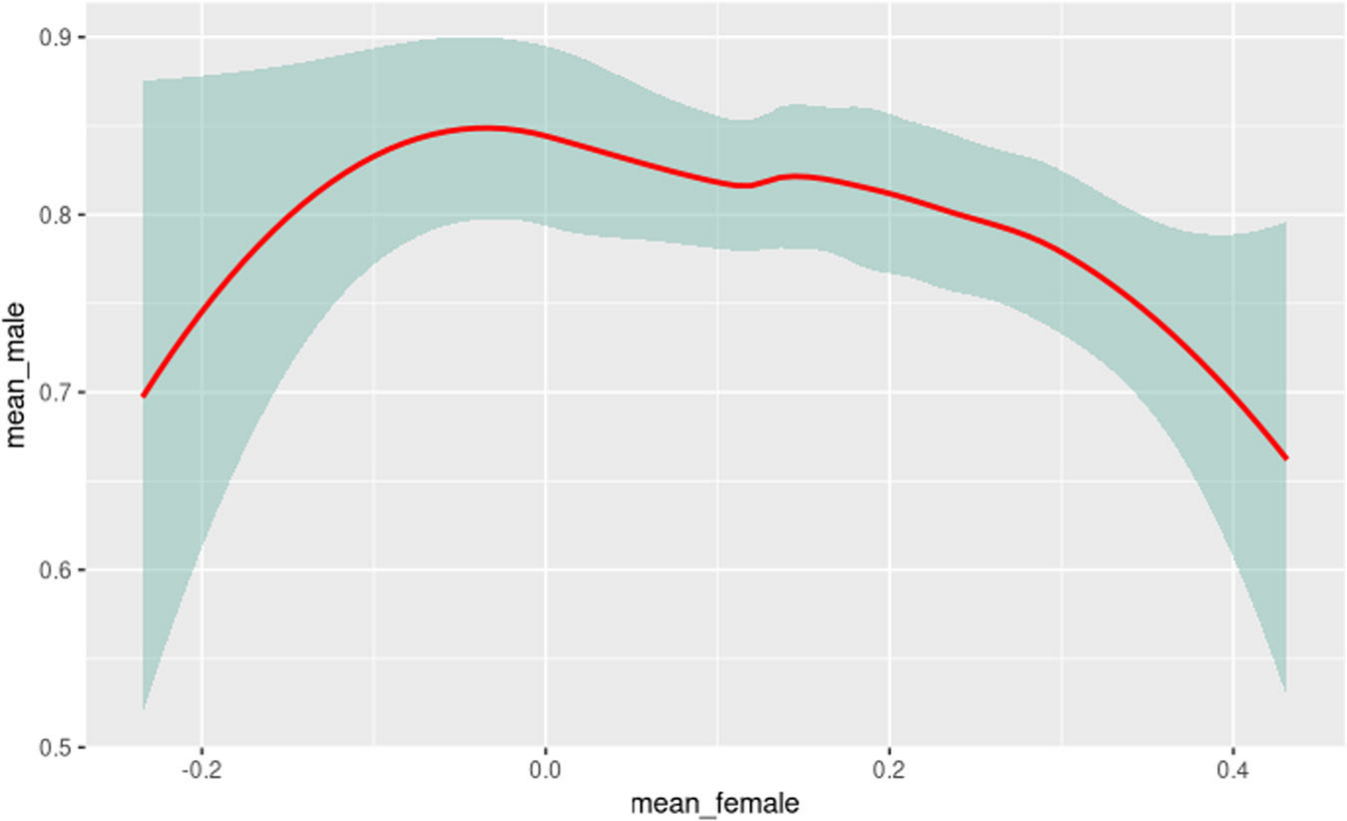
Variation of preference levels in their reliability intervals. The figure shows the variation of the correlation between colors with feminine biases of colors with masculine biases. When there is much male bias, the colors with female bias are almost nil. On the other hand, the higher the female colors, the lower the male color values.

The variation among male and female preference levels was plotted alongside its preference intervals (Fig. 8). The plots further confirmed the polarity of preference when low values were obtained in the female scale, while the highest values were found in the male scale. Consequently, when the female preference level tends to zero, the male preference levels would reach the highest values, and vice-versa.

Results of the robust correlation level among preference levels, as well as their statistical significance, were calculated considering the critical reliability value of 95% (Table 9). It is noticed that an inverse correlation reinforces the polarity or contrariwise proportion effect previously mentioned. Furthermore, the variation of levels in their respective reliability intervals indicated a weak-moderate effect of *−0.4947* taking into account the strength of correlation on standard scales. The *p-value* for this comparison was of *0.00002*, indicating a significant correlation in this analysis.

**Table 9:** Robust correlation between levels of male vs. female preference.

Robust correlation coefficient	Statistical *T*	*p*-value
−0.494	−4.796	<0.01

### Research Question 2: Color-bias in educational technologies by type

The color bias was also investigated to evaluate variations of preference level bias by educational technology type. As aforementioned in the descriptive statistic subsection, the technology types considered for this research were: (i) CMS—content management systems; (ii) RLE—remote learning environments (AVA—Ambientes Virtuais de Aprendizagem); (iii) Gamified Environments; and lastly (iv) MOOCs—Massive open online courses.

Results for technology types color bias were calculated separately for gender. For males, *p*-values (<0.001) presented statistically significant differences, indicating noteworthy differences among color bias in their technologies. A paired analysis using adjustment of denominated *p* post hoc *tests on the trimmed means* was conducted to highlight divergent technologies or those which possess high levels of preference bias (Table 10). Results indicated that CMS technologies displayed the highest male-oriented bias levels for colors inherent to the design, while RLE was the technology type with the lowest male color bias. Despite significant *p*-value for MOOC and gamified environments, the latter took second place among environments with the highest male color bias. The results also presented a 0.38 correlation value, indicating a weak to moderate relationship between technology types. However, while considering existing differences between color levels belonging to the male colors scale, it is necessary to verify the existence of female levels of difference. Still, as mentioned by distinct authors, the scales are not dichotomous and are not complementary. The results demonstrated that technology types with the highest female bias are RLE and CMS, followed by MOOC and, lastly, gamified environments (Table 11). Moreover, CMS and MOOC presented similar preference levels, representing non-significant *p* values (0.14434). The existing correlation between educational technologies’ colors that consider color preference for the female gender is also weak to moderate, with a value of 0.26.

**Table 10:** Robust one-way comparison for color bias and technology types: male bias.

Statistical *F*	*p*-value	Bootstrap CI	Effect size
31.725	<0.01	[0.27–0.47]	0.38
Post hoc tests on the trimmed means			
**Comparison**	**Lower CI**	**Upper CI**	**Adj.** ***p*-value**
rle vs. cms	−0.087	−0.043	<0.01
rle vs. gamified environment	−0.065	−0.022	<0.01
rle vs. mooc	0.058	−0.015	<0.01
cms vs. gamified environment	0.012	0.031	<0.01
cms vs. mooc	0.018	0.038	<0.01
gamified environment vs. mooc	−0.001	0.015	0.037

**Table 11:** Robust one-way comparison for color bias and technology types: female bias.

Statistical *F*	*p*-value	Bootstrap CI	Effect size
34.707	<0.001	[0.17–0.35]	0.26
Post hoc tests on the trimmed means			
**Comparison**	**Lower CI**	**Upper CI**	**Adj.** ***p*-value**
rle vs. cms	−0.006	0.038	0.111
rle vs. gamified environment	0.032	0.076	<0.01
rle vs. mooc	0.000	0.047	0.02
cms vs. gamified environment	0.026	0.049	<0.01
cms vs. mooc	−0.006	0.021	0.144
gamified environment vs. mooc	−0.043	−0.017	<0.01

### Research Question 3: color-bias in educational technologies by teaching subjects

The preference bias among educational technology contexts presented statistically significant differences for some of the contexts. The color preference belonging to the male scale indicated the highest male color bias is that of Computer Science, followed by Programming. On the opposite side, Business and Sciences presented the lowest male bias compared to the other contexts, with relatively the same male bias level. Technologies of Languages, Math, and Multidisciplinary contexts presented intermediary levels of male bias. Moreover, the two latter also presented similar levels, with non-significant *p* values (*p* = 0.38063) (Table 12).

**Table 12:** Robust one-way comparison for color bias and teaching subjects: male bias.

Statistical *F*	*p*-value	Bootstrap CI	Effect size
266.783	<0.001	[0.47–0.63]	0.55
Post hoc tests on the trimmed means			
**Comparison**	**Lower CI**	**Upper CI**	**Adj.** ***p*-value**
Business vs. computer science	−0.146	−0.107	<0.01
Business vs. languages	−0.099	−0.073	<0.01
Business vs. math	−0.079	−0.041	<0.01
Business vs. multidisciplinary	−0.077	−0.060	<0.01
Business vs. programming	−0.112	−0.094	<0.01
Business vs. sciences	−0.063	0.048	0.642
Computer science vs. languages	0.018	0.061	<0.01
Computer science vs. math	0.040	0.091	<0.01
Computer science vs. multidisciplinary	0.038	0.077	<0.01
Computer science vs. programming	0.004	0.043	<0.01
Computer science vs. sciences	0.061	0.176	<0.01
Languages vs. math	0.004	0.047	0.001
Languages vs. multidisciplinary	0.004	0.031	<0.01
Languages vs. programming	−0.030	−0.002	<0.01
Languages vs. sciences	0.022	0.135	<0.01
Math vs. multidisciplinary	−0.027	0.010	0.380
Math vs. programming	−0.062	−0.023	<0.01
Math vs. sciences	−0.005	0.110	0.016
Multidisciplinary vs. programming	−0.043	−0.025	<0.01
Multidisciplinary vs. sciences	0.005	0.116	0.005
Programming vs. sciences	0.039	0.151	<0.01

Statistically significant differences were also found in educational technologies by teaching subjects on the female scale (Table 13). Test results identified a correlation among levels to be considered from moderate to strong, with a 0.69 value. Technologies belonging to Business contexts presented the highest female preference levels, followed by the Sciences, which also presented the highest color variability on the female scale. On the other hand, technologies associated with the Computer Science context presented the lowest levels of female preference. Nevertheless, Programming was the third-largest context compared to other technologies within the female color scale level. Math, Languages, and Multidisciplinary contexts presented closely related color levels for the female gender.

**Table 13:** Robust one-way comparison for color bias and teaching subjects: female bias.

Statistical *F*	*p*-value	Bootstrap CI	Effect size
176.844	<0.001	[0.61–0.78]	0.69
Post hoc tests on the trimmed means			
**Comparison**	**Lower CI**	**Upper CI**	**Adj.** ***p*-value**
Business vs. computer science	0.263	0.326	<0.01
Business vs. languages	0.215	0.273	<0.01
Business vs. math	0.188	0.264	<0.01
Business vs. multidisciplinary	0.199	0.257	<0.01
Business vs. programming	0.153	0.213	<0.01
Business vs. sciences	−0.012	0.184	0.02
Computer science vs. languages	−0.068	−0.032	<0.01
Computer science vs. math	−0.100	−0.037	<0.01
Computer science vs. multidisciplinary	−0.084	−0.048	<0.01
Computer science vs. programming	−0.131	−0.092	<0.01
Computer science vs. sciences	−0.305	−0.111	<0.01
Languages vs. math	−0.046	0.010	0.102
Languages vs. multidisciplinary	−0.027	−0.004	<0.01
Languages vs. programming	−0.075	−0.047	<0.01
Languages vs. sciences	−0.255	−0.062	<0.01
Math vs. multidisciplinary	−0.025	0.030	0.787
Math vs. programming	−0.072	−0.013	<0.01
Math vs. sciences	−0.239	−0.041	<0.01
Multidisciplinary vs. programming	−0.059	−0.031	<0.01
Multidisciplinary vs. sciences	−0.239	−0.046	<0.01
Programming vs. sciences	−0.193	−0.000	0.010

### Research Question 4: color-bias in educational technologies by age group

The age-group analysis did not indicate significant differences between males (Table 14) considering technologies divided by their respective target groups or referring to their appropriate age groups. Therefore it is possible to infer that the technologies presented equivalent bias loads. In other terms, regardless of age group, educational technologies presented similar high values among target groups. Therefore, paired analyses were not conducted, given that the technologies were divided by their respective age groups and did not present statistically significant differences in male color bias.

**Table 14:** Robust one-way comparison for color bias and age group: male bias.

Statistical *F*	*p*-value	Bootstrap CI	Effect aize
0.297	0.879	[0.01–0.05]	0.03

In this analysis, females presented statistically significant differences between the age groups of these educational technologies concerning color level, with a *p*-value of <0.001 (Table 15), despite the weak effect size (0.11) in the scale. The paired comparisons were conducted with adjusted *p*-values to detect significant differences between female age groups. Results indicate differences among educational technologies for the 01–18 years old group, which presented the lowest female preference levels. The remaining technologies presented preference levels without significance, with equivalent color scales for age groups.

**Table 15:** Robust one-way comparison for color bias and age group: female bias.

Statistical *F*	*p*-value	Bootstrap CI	Effect size
23.060	<0.001	[0.09–0.14]	0.11
Post hoc tests on the trimmed means			
**Comparison**	**Lower CI**	**Upper CI**	**Adj.** ***p*-value**
01–18 vs. 19–24	−0.030	−0.013	<0.0001
01–18 vs. 25–35	−0.030	−0.013	<0.0001
01–18 vs. 36–50	−0.030	−0.013	<0.0001
01–18 vs. 51–69	−0.030	−0.013	<0.0001
19–24 vs. 25–35	−0.009	0.009	1
19–24 vs. 36–50	−0.009	0.009	1
19–24 vs. 51–69	−0.009	0.009	1
25–35 vs. 36–50	−0.009	0.009	1
25–35 vs. 51–69	−0.009	0.009	1
36–50 vs. 51–69	−0.009	0.009	1

## Discussion

The discussion is centered around answering, discussing, and pointing out the effects and results produced and presented in the previous section to facilitate the comprehension of the results, aligned with the hypotheses of this research.

Therefore, the null hypothesis was rejected, resuming the first research question, which investigated the existence or not of a color bias in educational technologies. Results indicated **H**_1.1_“statistically significant differences between color levels in educational technologies”. The results show an overall male-oriented bias toward colors in the design of educational technologies. One point that raises attention is that currently, women are still a minority in technology courses. Some studies further discuss this gender imbalance (Cheryan et al., 2017; Shein, 2018; Stevenson, 2020), and these report males as the majority in these areas. This imbalance could consist a significant influence factor in the development of educational technologies, which are often strongly biased towards the male gender. Another reason may be the groups responsible for developing these technologies, which could be imbalanced and composed mainly of males. According to the American Computer Science Association^4^, women represent 18% of the students who graduate in computer science. Furthermore, women sum up to 37% of the students in undergraduate programs belonging to the STEM fields (science, technology, engineering, and mathematics; Cheryan et al., 2017).

When observing the results for research question 2 (color bias in educational technologies by type), the null hypothesis was rejected, indicating the presence of **H**_2.1_ “statistically significant differences between the color levels in educational technologies by type”. Thus, the presence and dilution of attributes related to each technology’s color bias and design elements are identified. Correlating results for the male scale, gamified environments presented the lowest bias levels for the female scale and a high male bias. Therefore, it is logical to raise assumptions that advantages for the male gender in diverse aspects referring to their colors are present in these environments. In turn, content management systems (CMSs)—systems built exclusively for content management, presented colors tied to the solution archetype and its respective educational resources. The student’s follow-up is even higher since it is a presentation and content exhibition of educational technology. According to De la Varre et al. (2014), about evasion, these system modality flaws are related to the lack of mediation from tutors and teachers. These flaws can be classified as a potential problem for opposite-gender students in this type of technology due to the heavily influential role of color bias.

Concerning the third research question, which aimed to observe the color bias in the context of educational technologies, the results showed that **H**_3.1_ “statistically significant differences between the color levels in educational technologies by context”. Hence, rejecting the null hypothesis. Nonetheless, the technology context with the highest level of male color bias and the lowest level of female bias was Computer Science. Once again, since women in this field of study can be considered a minority (as in STEM fields generally), can this bias be a fundamental factor for women’s disinterest and evasion rates in this course modality? Some studies discuss representativeness and mediators such as anxiety of women in these courses (Camp, 2002; Nicolai, 2001) or overall personal interests?. Authors further discussed anxiety through stereotype threat in educational technologies in the performance in logic activities (Albuquerque et al., 2017). Thus, relating the aspect mediated by color interference to the emergence of possible stereotype threats in educational technologies could generate anxiety and further reinforce this issue for female students.

Finally, the results of the fourth research question, color bias in educational technologies by age group (**H**_4_), showed two strands for each age group. The first strand did not reject the null hypothesis for male color bias, and the second rejected the null hypothesis for female bias. The literature on color psychology and their preferences identifies that each age and gender presents a certain level of preferred colors. While divergence of colors can be based on gender, obtained through the scales used in this study, different age groups might present it as well (Hallock, 2003). It is possible to raise some assumptions about the current study results. The first is that technologies present the same level of color bias for males, implying that males do not shift in color preference as much. The second is the lack of standardization in the elaboration of technologies for colors belonging to the female gender, with age not being a factor taken into consideration.

## Conclusion

The presented and discussed results in this study align with the current literature. Despite both scales being independent, the results present evidence of the strong predominance of colors belonging to the male scale in these evaluated technologies. In other terms, educational technologies are elaborated with a strong bias toward the male gender. This bias can be related to the more significant number of male students who graduate in the listed fields of the study compared to the number of female students who seek universities or further education in these areas.

Nevertheless, the development of technologies that consider the possibility of color customization is still limited. Different technologies, regardless of the type and applied context, present low variance in color use when compared to each other. Furthermore, based on our results, gender should be a factor of utmost importance to make educational technologies more inclusive and egalitarian. This limitation is perhaps an associated cause of the evasion of female students in the STEM fields.

Despite independent preferences in the scales, it was possible to observe a dichotomy between colors, reinforcing the opposite effect of gender-related preferences. The existing correlation between male and female colors showed a moderate negative effect, indicating an opposite effect to the effect observed.

### Limitations, threats to validity and future works

This study comprised only 73 educational technologies collected randomly, with 3136 pages from the WEB. With their respective ages, the target group could be better defined if more precise information was available on the educational technology’s websites. Moreover, the number of users was estimated based on the report for some technologies, which can indicate an inaccuracy of the number of students, indicating only the number of registered students. We acknowledge that while there can be cases of more than one student using the same profile, there is also the possibility of students having more than one profile, thus causing variation in the actual number of users.

In the future, we plan to expand this study aims and collect data to observe the effect of textual elements also extracted from the educational technologies to analyze negative stereotypes contained in the textual content. Furthermore, future work is intended to improve analysis towards age group, considering the preference scale in this study. Additionally, we intend to increase the dataset generated in this study to build models to use artificial intelligence capable of predicting male and female color bias.

## Data Availability

The datasets analyzed during the current study are available in the Dataverse repository: 10.7910/DVN/NA0US1.

## Footnotes

1 shorturl.at/ghvyI

2 Science, Technology, Engineering, and Math.

3 Quantiles are points belonging to regular intervals from the accumulated distribution function of a random variable. The quantiles divide the sorted data into **q** data subsets of essentially equal dimensions.

4 https://www.computerscience.org/resources/women-in-computer-science/
